# Potential Activity of Aqueous Fig Leaves Extract, Olive Leaves Extract and Their Mixture as Natural Preservatives to Extend the Shelf Life of Pasteurized Buffalo Milk

**DOI:** 10.3390/foods9050615

**Published:** 2020-05-11

**Authors:** Mohamed El Dessouky Abdel-Aziz, Mohamed Samir Darwish, Azza H. Mohamed, Ayman Y. El-Khateeb, Sahar E. Hamed

**Affiliations:** 1 Dairy Department, Faculty of Agriculture, Mansoura University, Mansoura, Mansoura 35511, Egypt; meldosoky@mans.edu.eg (M.E.D.A.-A.); msamir@mans.edu.eg (M.S.D.); 2 Agricultural Chemistry Department, Faculty of Agriculture, Mansoura University, Mansoura 35511, Egypt; aymanco@mans.edu.eg; 3 University of Florida, IFAS, Citrus Research & Education Center, 700 Experiment Station Road, Lake Alfred, FL 33850, USA; 4 Chemistry Department, Faculty of Agriculture, Damietta University, Damietta 34517, Egypt; s.hamed@du.edu.eg

**Keywords:** pasteurized buffalo milk, olive leaves, fig leaves, aqueous extracts, antioxidants and antimicrobial agents

## Abstract

The aim of this study was to evaluate fig (*Ficus carica* L.) leaves’ extract (FLE), olive (*Olea europaea* L.) leaves’ extract (OLE), and their mixture (MLE), to extend the shelf life of pasteurized milk. OLE, FLE, and their mixture MLE (1:1) were added to the pasteurized milk in different concentrations (0.2%, 0.4%, and 0.6%). Several tests were then conducted to determine the activity of these extracts. The antioxidant activity as IC_50_ was determined by using DPPH radical assay. FLE showed higher IC_50_ (30.21 µg/mL) compared to the IC_50_ of OLE (22.43 µg/mL). Phenolic compounds were identified by using high-performance liquid chromatography (HPLC). The highest antimicrobial activity was obtained with 0.6% concentration. Organoleptic properties indicated that the addition of these extracts did not affect the sensory properties of pasteurized milk. Pasteurized milk treated with 0.6% of FLE, OLE, and MLE has significantly decreased (*p* ≤ 0.05) lipase and protease activity during the storage period, at 5 °C. The results indicated that extending the shelf life of pasteurized milk from 5 to 16 days was successfully achieved through using 0.6% of FLE, OLE, and MLE. The combination of the two extracts (MLE) provides an efficient and safe method to prolong the shelf life of pasteurized milk, without altering the properties of pasteurized buffalo milk.

## 1. Introduction

There are a lot of human diseases associated with many foods, as a result of foodborne bacterial pathogens, which is a global interest with a significant effect on consumer health. Access to the appropriate quantities of nutritious and safe food is a critical point to enhance human health and to sustain life. Contaminated foods may lead to a high number of illnesses. These so-called foodborne diseases (FBD) resulted from various agents, such as viruses, bacteria, toxins, chemicals, and parasites, and lead to several clinical symptoms [1]. Reports showed that about 420,000 people die every year all over the world due to food contamination. Moreover, a lot of people get sick each year, with 40% of children under the age of five getting sick from foodborne diseases [1]. Diarrheal maladies are the major common diseases caused by contaminated food consumption, resulting in 550 million individuals to be sick and 230.000 deaths each year.

The high nutritional value of raw milk leads to microbial proliferation that becomes the leading cause of milk-borne diseases and can trigger milk spoilage [2]. The spoilage of raw milk is directly associated with the existence of *Pseudomonas* sp., *Bacillus* sp., and *Micrococcus* sp. Furthermore, the presence of pathogenic microorganisms such as *Escherichia coli, Salmonella* Typhi, *Shigella sonnei*, *Listeria monocytogenes,* and *Campylobacter jejuni* caused milk-borne diseases [2,3]. Therefore, foodborne bacterial pathogen control is a primary concern for the food industry and regulatory agencies.

The existence of foodborne pathogens in milk can generally occur from different sources, for example, from the interior udder infection, the exterior of the udder, and from the equipment surface used for milk handling and storage [4]. Several milk-borne epidemics of human illnesses have been spread by milk contamination by dairy workers’ dirty hands, contaminated water supplies, flies, and unsanitary utensils [4]. Milk and dairy products provide a primary source of different microorganisms and, therefore, are considered one of the essential causes of foodborne pathogens. Foodborne illness pathogens present in milk can be due to outbreaks related to cow milk and dairy products’ consumption were detected to result in 761 diseases and 22 hospitalizations per year in the USA. Raw milk and unpasteurized products are used by a few American consumers but lead to 95% of sick conditions. The risk for the disease was detected to be more than 800 times greater for the consumer of raw milk and unpasteurized dairy products than for the consumer of pasteurized dairy products. The outbreak caused by consuming dairy products is usually driven by campylobacteriosis and salmonellosis [5].

In milk and dairy products, to ensure the complete elimination of pathogenic microbes, in addition to other non-pathogenic spoilage microbes’ reduction and inactivation of undesirable enzymes, a highly efficient process is required. The most effective procedures are ultra-high temperature (UHT) and pasteurization [2]. Using ultra-high temperature provides a remarkably long shelf-stable milk (up to 8–12 months) when kept at room temperature. On the other hand, this procedure causes a significant loss of the flavor, color, several vitamins, and antimicrobial compounds [6,7,8]. However, pasteurized milk is a high-nutritional-value product, but with a limited shelf life (only 5–7 days) at 5–8 °C, and it is costly compared with UHT milk [9]. The pasteurization techniques of milk are divided into two common types, according to the Codex Alimentarius [10]. The first type is the low-temperature long time (LTLT) at 62.5 °C for 30 min, which is recommended for batch pasteurization. The second type is the high-temperature short time (HTST) at 72 °C for 15 s, which is required for continuous pasteurization [10]. The effect of the milk pasteurization process is limited to eliminating only pathogenic microbes, but its impact on thermoduric bacteria and spores is lacking [2]. The main disadvantage of pasteurization is associated with a limited shelf life of pasteurized milk [2]. The quality of pasteurized milk changes over time during storage by spoilage microbes, especially bacterial sporeformers, such as Bacillus sp. [11]. This bacterium has the ability to produce lipase, phospholipase, and proteinase, thus producing off-flavors (bitty cream and sweet curdling defects) [11]. One of the methods in which foodborne bacterial pathogens can be controlled is by using food preservatives. These preservatives can be divided into natural antibacterial compounds and synthetic chemicals. The most important part of natural antimicrobial compounds as food preservatives is plant antimicrobials. Plant antimicrobials receive more attention, since they are generally considered very safe and have many positive effects on human health [12]. Recent scientific researches indicated the potential use of natural antimicrobials with a broad spectrum of antimicrobial effect that may be employed to extend perishable foods’ shelf life [13,14]. Some possible beneficial plant antimicrobials have been characterized [15,16,17]. Along with the newest health and food safety standards, the demand for replacing chemical preservatives with natural compounds has increased. The most important strategy to maintain the high quality of pasteurized milk and extend the expiration date is through a complementary stage with naturally recognized antimicrobial compounds, such as olive leaves’ extract (OLE) and FLE. Many studies reported the antibacterial efficiency of OLE or its phenolic compounds against several bacterial species, including *B. cereus* [18,19], in addition to its effectiveness in extending the shelf-life period of foods [20,21,22]. OLE also has positive effects on public health, such as antihypertension, and supporting the immune and cardiovascular systems and increasing the energy levels [23,24,25]. Olive oil is composed mainly of triacylglycerols (triglycerides or fats) and contains small quantities of free fatty acids (FFA), glycerol, phosphatides, pigments, flavor compounds, sterols, and microscopic bits of olive. Triacylglycerols are the major energy reserve for plants and animals. Olive leaves are considered as a potential inexpensive source for food supplements for human health [26]. Phenolic compounds in olive leaves are major contributors to their antioxidant effect [27]. According to Acar-Tek and Ağagündüz [28], the toxicity studies suggest that olive leaf is generally safe, even at high doses.

The leaves of *Ficus carica* (FLE) have health benefits involving antidiabetic activity [29]. The FLE and fig fruits are utilized to treat throat diseases. Moreover, it is used as a laxative, stimulant, emollient, antitussive, resolvent, and emmenagogue [30]. The FLE and latex included approximately 91% of the active compounds leading to the antimicrobial activity against several pathogenic bacteria [31]. OLE and FLE can be used as natural preservatives, to control food-poisoning diseases [14].

Under Egyptian regulation, there are no rules against the addition of plant-derived natural antimicrobials or the maximum concentration of the natural antimicrobials into foods. The National Food Safety Authority (NFSA) will publish a special dietary foods list, nutritional supplements, and food for particular medical uses that are undergoing registration under the recent regulation [32]. Therefore, the purpose of this study was to evaluate fig (*Ficus carica*) leaves’ extract (FLE), olive (*Olea europaea*) leaves’ extract (OLE), and their mixture (MLE), to extend the shelf life of pasteurized milk.

## 2. Materials and Methods

### 2.1. Reagents and Chemicals

The chemicals and solvents included DPPH, ethyl alcohol, Na_2_CO_3_, NaOH, Fehling’s A solution, Fehling’s B solution, amyl alcohol, and H_2_SO_4_, in addition to all standards, reagents, and HPLC reagents; all were obtained from Sigma-Aldrich company (Cairo, Egypt).

### 2.2. Source of Milk

Fresh whole buffalo milk was obtained from the Center for Agricultural Research and Experiments, Faculty of Agriculture, Mansoura University, Mansoura, Egypt. The experiment was done in the laboratory of the Dairy Products Department of the Faculty of Agriculture at Mansoura University.

### 2.3. Plant Materials

Olive (*Olea europaea*) and fig (*Ficus carica*) leaves were obtained from a private farm located at El-Arish City, North Sinai Governorate, Egypt, in August 2018. Olive (*Olea europaea*) leaves were collected from Manzanillo olive trees’ cultivar. Leaves required in this study were randomly sampled from shoots from four directions in each tree out of thirty trees (15-year-old trees). Two or three leaves within every shoot (4th and 5th leaves) were collected, and then all leaves were combined for performing the subsequent steps in this study. Fig (*Ficus carica*) leaves were collected from Sultani fig trees’ cultivar. Leaves sampling was done at the beginning of fruit production; newly mature leaves were collected from the middle portion of branches from different sides of the fig trees which had appropriate sun exposure. Leaves sampling was from thirty trees, four leaves per tree. Then all leaves were combined and used for the following steps. The leaves were then sent directly to the Dairy Products Department of the Faculty of Agriculture at Mansoura University. The fresh leaves were washed and dried at 45 °C, until constant weight, and subsequently ground in a Braun GmbH grinder (KSM2; type, 4041). They were then sieved with a 75–100 μm mesh sieve, according to Ibrahim et al. [33]. The powder was stored in a dark container for later use.

#### Preparation of Aqueous Extracts of Olive and Fig Leaves 

The plant powder of fig leaves and olive leaves (4 g of finely ground leaves’ powder suspended in 96 mL deionized water at 75 °C for 15 min) was prepared as illustrated by Palmeri et al. [34]. The FLE and OLE were filtered by sterilized membrane filter (0.20 µm pore-size). Then the filtrates were concentrated by using a rotary evaporator at 50 °C, followed by drying in an oven at 50 °C [34].

### 2.4. Chemical Analysis of the Examined Leaves

The chemical analysis of fig leaves and olive leaves was done to detect the contents of moisture, ash, crude protein, crude fat, and crude fiber on weight dry (DW), using 6.25 as a factor for protein calculation. Moreover, total and soluble carbohydrates were calculated by difference, using the following equations, according to AOAC [35]:

% Total Carbohydrates = 100 − (ash + crude protein + crude fat + crude fiber)

% Soluble Carbohydrates = Total carbohydrates − Crude fiber

### 2.5. Determination of Minerals Content

Ashes of the plant materials (fig leaves and olive leaves) were put in 1 mL of concentrated HCl and dissolved completely. The volume was completed to 100 mL with distilled water. Different minerals in the solution were then detected by the suitable method. Potassium was detected by using a flame photometer according to Hesse [36]. Magnesium, calcium, and manganese were detected as described by Cottenie et al. [37], using an atomic absorption instrument (model, Perkin-Elmer 2380). Phosphorus was measured calorimetrically according to Page [38].

### 2.6. Quantitative Assessment of Total Polyphenol Level

The total phenolic content of OLE and FLE was determined by mixing the extract (0.1 mL) with precisely 2.8 mL of distilled water and sodium carbonate (2.0 mL, 2% *w/v*), and finally the Folin-Ciocalteu reagent (0.1 mL, 50% *v/v*). The incubation of the mixture was done at room temperature, for 30 min, in the dark. The absorbance of the resulted color was measured by using a UV spectrophotometer at 750 nm. The standard curve of gallic acid (0–200 mg L^−1^) was prepared. The total content of phenolic compounds was calculated by using the Gallic acid calibration curve and expressed in milligrams of gallic acid equivalent (GAE)/g of dry weight, according to the method described by Ibrahim, EL-Khateeb, and Mohamed [33].

### 2.7. Preliminary Phytochemical Determination of the Aqueous Leaves’ Extracts

The qualitative tests were done on the aqueous extracts of plants, to detect the presence of flavonoids, steroids, tannins, saponins, and alkaloids, according to Thilagavathi et al. [39].

#### 2.7.1. Detection of Flavonoids

Leaves’ extract (4 mL) was macerated in HCl (1%) overnight, followed by the addition of a solution of NaOH (10%) to the filtrate. The appearance of a yellow color indicates the presence of flavonoids [39].

#### 2.7.2. Detection of Steroids

The test was carried out according to Thilagavathi, Rajasekar, and Doss [39]. First, 2 mL of leaves’ extract was transferred to a clean and dry test tube. Chloroform (10 mL) was added, followed by 1 mL of acetic anhydride. The mixture was shaken to be mixed well. After that, 2 mL of concentrated H2SO4 was added slowly and carefully to the sides of the test tube. The appearance of a blue–green color at the junction indicates the presence of the steroids in the plant extract.

#### 2.7.3. Detection of Tannins

First, 2 mL of leaves’ extract was transferred to a clean and dry test tube, and then a few drops of 10% lead acetate was added. The appearance of a white precipitate indicates the presence of tannins [39].

#### 2.7.4. Saponin Detection

Distilled water (9 mL) was added to 1 mL of the leaves’ extract in a measuring cylinder. Then, the mixture was stirred strongly for at least 15 s. After that, the mixture was allowed to stand at room temperature for 10–15 min. The appearance of the foam layer (1 cm) on the surface indicates the presence of saponins in the plant extract [39].

#### 2.7.5. Detection of Alkaloids

Leaves extract (2 mL) was mixed with a few drops of diluted HCl. The solution was then filtrated, and 1 mL of Dragendorff reagent was added. The orange-to-red precipitate indicates the presence of alkaloids [39].

#### 2.7.6. Glycosides Detection

A few drops of glacial acetic acid, FeCl_3_, and 3–4 drops of concentrated H_2_SO_4_ were added to 1 mL of the leaves’ extract. The blue–green color indicates the presence of glycosides [39].

### 2.8. Fractionation and Identification of Phenolic Compounds

Phenolic compounds were identified by using high-performance liquid chromatography (HPLC) analysis. This analysis was done at Food Safety and Quality Control (FSQC) Laboratory, Faculty of Agriculture, Cairo University, Egypt, using Agilent 1260 infinity HPLC Series (Agilent, Tampa, Florida, USA), equipped with quaternary pump, aKinetex^®^5μm EVO C18 (100 mm × 4.6 mm), Phenomenex, Tampa, Florida, USA, operated at 30 °C. The separation was done by a ternary linear elution gradient with (A) HPLC grade water 0.2% H3PO4 (*v/v*), (B) methanol, and (C) acetonitrile. Then, 21 external standards which were used for quantitation determination. For more information, the characteristics and performances of the calibration curves of the individual reference standards are presented in Supplementary Table S1. The injection volume was 20 μL, and the VWD detector was set at 284 nm. All standard polyphenols obtained from sigma Co. were dissolved in the mobile phase and injected into the HPLC instrument. Retention time and peak area were used to calculate the concentrations of phenolic compounds content by analyzing the data of HP software according to Yang et al. [40].

### 2.9. Antioxidant Activity and the IC_50_ of OLE and FLE Using DPPH Radical Assay

The antioxidant activity was done by using the DPPH trapping capacity of each concentration OLE, and FLE extracts were established as described by Ibrahim, EL-Khateeb, and Mohamed [33]. The content of DPPH radical removal activity was calculated according to the following equation:

(1) $$\%\;\mathrm{Scavenging}\;\mathrm{activity}\;\left(\%\;\mathrm{RSA}\right)=\frac{\mathrm{Absorbance}\;\mathrm{blank}-\mathrm{Absorbance}\;\mathrm{sample}}{\mathrm{Absorbance}\;\mathrm{blank}\;}\times 100$$

The scavenging activity was plotted against concentration and IC_50_ (the extract concentration providing 50% of radicals scavenging activity) value of DPPH was calculated from the graph by linear regression analysis.

### 2.10. The Preliminary Evaluation of the Antimicrobial Activity of OLE, FLE, and MLE

The potential antimicrobial activity of OLE, FLE, and MLE against foodborne pathogens involving three Gram-positive bacterial strains (*Staphylococcus aureus*, *Enterococcus faecalis*, and *Bacillus cereus*) and three Gram-negative bacterial strains (*Escherichia coli*, *Salmonella enterica serovar* Typhi, and *Pseudomonas aeruginosa*) was generally tested by the agar diffusion method, as described by Hsouna, et al. [41]. This test was done in the microbiology laboratory at the Dairy Department, Collage of Agriculture, Mansoura University, Mansoura, Egypt.

### 2.11. Preparation of Aqueous-Extracts-Enriched Pasteurized Milk 

**Figure 1:**
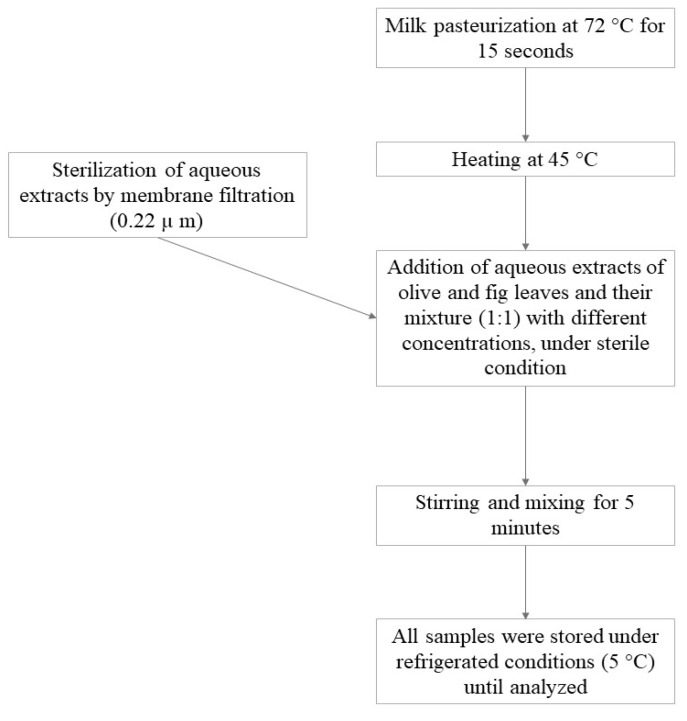
Pasteurized buffalo milk enriched with different concentrations of aqueous leaves’ extracts (olive and fig and their mixture “1:1”).

The preparation of different concentrations of aqueous extracts of OLE, FLE, and MLE (the ratio of FLE to OLE was one to one). The enriched pasteurized milk was prepared according to Brand-Williams et al. [42], with slight modification, as described in Figure 1.

### 2.12. The Microbiological Analysis of FLE, OLE, and MLE Enriched Pasteurized Milk

#### 2.12.1. Determination of Total Aerobic Counts (TAC)

The procedure used to determine TAC in FLE-, OLE-, and MLE-enriched pasteurized milk is described in ISO method 4833-1 [43].

#### 2.12.2. Determination of Total Psychrotrophic Counts (TPC)

The procedure used to determine TPC in FLE-, OLE-, and MLE-enriched pasteurized milk is described in ISO method 6730 [44]. 

#### 2.12.3. Determination of the Psychrotrophic Aerobic Bacterial Spore Counts (PABSC)

The procedure used to determine PABSC in FLE-, OLE-, and MLE-enriched pasteurized milk is described in APHA protocol 8-090 [45].

#### 2.12.4. Determination of Enterobacteriaceae Count (EC)

The procedure used to determine EC in FLE-, OLE-, and MLE-enriched pasteurized milk is described in ISO 21528-2 [46].

### 2.13. Measurement of pH Value and Acidity 

The pH meter (digital pH meter, Hanna instruments, the HI2020 edge^®^) was used to measure the pH of the tested samples [47]. The titratable acidity of FLE-, OLE-, and MLE-enriched high-temperature short term (HTST) milk was determined after periodic intervals of storage at 5 °C. A 10 mL sample of milk sample was transferred to the conical flask, followed by 4 drops of phenolphthalein to sample, and titrated with 0.1-N sodium hydroxide; the endpoint of this reaction was determined by the change of milk color to pink color that remains constant for 15 s. The acidity was expressed as a lactic acid percentage [47].

### 2.14. Proteolytic Activity

Approximately 24 mg of Azocoll was transferred into a screw-capped test tube and was mixed with 4.5 mM KH2PO4 buffer (pH 7.5), followed by incubation for 5 min at 36 °C. Then, 0.5 mL of pasteurized milk was mixed for 45 s and was incubated at 36 °C for 30 min. The screw-capped tubes were then put on ice in an ice box, to stop the reaction. The mixture was then filtrated by using Whatman No.4 filter papers into a new screw-capped tube. The released azo dye absorption was measured at 520 nm against the blank, using a UV spectrophotometer. The protease activity was calculated by dividing the absorption of the developed color at 520 nm by 1/2.965, to produce the amount (mg) of hydrolysis of Azocoll at 37 °C in 25 min per test sample volume. The activity of protease was expressed in U/mg [48].

### 2.15. Lipolytic Activity

Free fatty acids (FFA) were determined by mixing 50 mL of ethanol with 10 mL pasteurized milk, and then 1 mL of phenolphthalein (1%) was added. The mixture was then titrated by using potassium hydroxide (1N). The endpoint of this reaction was determined by the change of milk color to a pink color that remains constant for 15 s [49]. The Free fatty acids concentration was calculated from the following equation:

(2) $$µ\mathrm{equiv}:\frac{\mathrm{FFA}}{\mathrm{mL}}=\left(\frac{T\;\times \;N}{P\;\times \;V}\right)\times \;1000$$

where *T* is the titration volume; *N* is the KOH normality; *P* is the titration volume; and *V* is the milk volume.

### 2.16. Analysis of Sensory Properties

The analysis of sensory characteristics of pasteurized buffalo milk samples was performed by 50 panelists (20 males; 30 females). The ages of recruits ranged between 20 and 45 years old; the volunteers were students and staff of the Faculty of Agriculture at Mansoura University. The highest scores were 50 points for flavors, 40 points for body and texture, and, finally, 10 points for appearance [50].

### 2.17. Statistical Analysis

Statistical analysis was done by using the SAS package [51]. Variance analysis (ANOVA), in a sense, was applied to compare the samples and tested treatments [51].

## 3. Results and Discussion

### 3.1. Chemical Composition of Olive and Fig Leaves

The chemical composition of olive and fig leaves is presented in Table 1. The obtained data showed that olive and fig leaves are a rich source of carbohydrates, protein, and ash. The moisture content in the olive leaves (8.12%) is lower than that moisture content in fig leaves (9.57%). Slight differences were observed regarding the lipid and ash contents between the olive and fig leaves. Olive leaves are higher in crude protein content (12.38%) than those obtained from fig leaves (7.22%), respectively. Similar data were also found by Cavalheiro et al. [52].

### 3.2. Minerals Content of Olive and Fig Leaves

The data presented in Table 2 illustrate the minerals content of olive and fig leaves. Olive and fig leaves are considered rich sources of minerals. A higher content of phosphorus, iron, magnesium, and manganese were detected in fig leaves, compared with the olive leaves. The calcium and potassium levels in olive leaves (1570 and 660 mg/100 g, respectively) are higher than the corresponding concentrations in fig leaves (1400 and 118.47 mg/100 g dry weight (DW), respectively). Calcium was found to be the main ash source in olive and fig leaves. The results revealed that calcium was the predominant element in the olive and fig leaves (1570 and 1400 mg/100 g DW, respectively). These results are consistent with Ibrahim et al. [53].

### 3.3. Phytochemical Screening of Aqueous Extract of Olive and Fig Leaves

Preliminary phytochemical screening of the aqueous extract of olive and fig leaves presented the existence of flavonoids, steroids, alkaloids, and tannins (Table 3). Saponins were not detected in OLE but were detected in FLE. The obtained results were expected, as compared with the results obtained by Adebisi and Oyeleke [54] and Liu et al. [55]. The obtained results came in agreement with Ahmed et al. [56]. Meanwhile, the existence of flavonoids, tannins, and saponins were detected in FLE [57]. However, the methanolic FLE was rich in terpenes, tannins, flavonoids, saponins, alkaloids, carbohydrates or glycosides, phenolic glycosides, and resins [58].

The content of bioactive compounds (flavonoids, steroids, tannins, saponins, and alkaloids) was directly proportional to the antioxidant and antimicrobial activities. As expected, the two extracts had antioxidant and antimicrobial properties due to their richness in bioactive compounds. This result is in agreement with the results reported by Liu, McKeever, and Malik [55].

Several probable mechanisms of action have been suggested for polyphenols as a response to the envelope of the pathogens. The mood of action is based on damage to the enzymatic processes involved in energy production during finishing or destruction of the permeability block of the cell membrane by varying the physiological state of the cells or affecting synthesis of the structural components [59]. The secondary metabolites of plants have a promising perspective as a source of effective antifungal agents, such as compounds derived from plants. These components, including hydroquinones, naphthoquinones, alkaloids, and flavonoids, have shown various antimicrobial activities [60].

### 3.4. Total Phenolic Compounds Levels (mg/g) and Antioxidant Activity (IC_50_) of Olive and Fig Leaves’ Aqueous Extracts

Free radicals are proven to play a critical role in a variety of pathological agents (included in several chronic and acute syndromes in humans). Antioxidants inhibit free radicals and defend from many progressive diseases [61]. The large molecule of the antioxidants (superoxide dismutase or catalase, etc.) absorbs reactive oxygen species (ROS) and prevents them from attacking other important proteins in the cell. However, the small antioxidant molecule (carotenoids, phenolics, tocopherol, ascorbic acid, and glutathione) neutralizes ROS in a way called free-radical scavenging. In the present study, the antioxidant activities of aqueous extracts of olive and fig leaves were estimated by 2,2-diphenyl-1-picrylhydrazyle (DPPH) and expressed in IC_50_ (the effective level of extract necessary to prohibit 50% of the initial DPPH). A higher IC_50_ value means a lower antioxidant activity of the plant extract. The IC_50_ of the aqueous extract of fig leaves was 30.21 µg/mL, which is higher than the IC_50_ of the aqueous extract of olive leaves (22.43 µg/mL), as shown in Table 4. The presented results of DPPH determination suggested that the FLE and OLE extracts had high antioxidant activity. These results are in agreement with other previous reports [62,63]. The high antioxidant activity of FLE and OLE might be because of the richness of the secondary metabolites, such as flavonoids, alkaloids, and polyphenols. Data representing the quantitative analysis of total polyphenols expressed in (mg of gallic acid equivalent/g of extract) of aqueous extracts of olive and fig leaves are illustrated in Table 3. The higher polyphenol content of 387 mg of gallic acid/g was found in the olive leaf extract, compared with 224 mg of gallic acid/g of extract in fig leaf extract. The content of plant phenolic compounds plays an essential role in the antioxidant effect of the two extracts. The total phenolic concentration of the aqueous extract of olive and fig leaves came in agreement with those reported by Adebisi and Oyeleke [54].

The suggested hypotheses in this study was that both FLE and OLE have a significant concentration of phenolic compounds (Table 4). These results were consistent with the results found in previous studies on both FLE and OLE [64,65]. Based on the previous studies, the results from the present study suggest a correlation between high phenols content and antioxidant activity [66]. Phenolic ingredients are the essential factors that contribute to the antioxidant activities of plant extracts [66]. Furthermore, the antiradical properties of phenolic components are due to their ability to bind with the free radical by donating their electrons, which are then converted into the stable state [67].

### 3.5. Characterization of the Phenolic Compounds by HPLC

Twenty-one phenolic compounds of FLE and OLE were characterized and quantified by HPLC (Table 5). The phenolic profile involved 15 phenolic acid derivatives, three free flavonoids, two simple phenols, one glycoside-phenol, and one tyrosol. Caftaric acid was characterized as the main phenolic compound in FLE, as 40.2 mg/g dried extract followed by quercitin (13.4 mg/g dried extract). The order of phenolic compounds based on the most abundant component in this study was as follows: p-hydroxy benzoic acid > caffeic acid > gallic acid (Table 5). Oleuropein was identified as the major phenolic compound in OLE as 32.2 mg/g dried extract, followed by ligstroside (4.2 mg/g dried extract) (Table 5). The HPLC results for the phenolic compounds of FLE were similar to the results reported by Nadeem and Zeb [68], who stated that caftaric acid, quercetin-3, 7-diglucoside, and quercetin-3-glucoside were the main phenolic compounds. The levels of phenolic compounds in OLE in the present study are consistent with those of Palmeri et al. [9], who showed that all phenolic compounds in OLE classified as polyphenols. As widely presented in the literature; oleuropein was 90% of phenolic compounds, followed by ligstroside (1.12 mg/g dried extract) and luteolin 7-O-glucoside (0.81 mg/g dried extract).

### 3.6. The Antimicrobial Activity of OLE, FLE, and MLE

The different concentrations of FLE, OLE, and MLE were used to examine their antimicrobial activity against foodborne pathogens. This experiment involved three Gram-positive bacterial strains (*Staphylococcus aureus*, *Bacillus cereus,* and *Enterococcus faecalis*) and three Gram-negative bacterial strains (*E. coli* O157:H7, *S.* Typhi, and *P. aeruginosa*), using the well diffusion agar method. Antimicrobial activity evaluation of extracts with different concentrations was recorded in Table 6. These results showed that FLE, OLE, and MLE were potentially useful in inhibiting bacterial growth of indicator microorganisms with variable potencies (Table 6). FLE was the most active extract inhibiting bacterial growth of Gram-negative bacterial strains (*E. coli, S. typhi,* and *P. aeruginosa*), as well as *Staphylococcus aureus* at a concentration of 0.6%. FLE has a relatively weak antimicrobial effect against both spores forming bacteria (*Bacillus cereus*) and thermoduric bacterial strain (*Enterococcus faecalis*). However, OLE was a very effective agent against Gram-positive bacterial strains (*Bacillus cereus, Enterococcus faecalis,* and *Staphylococcus aureus*). On the other hand, OLE showed relatively low activity against Gram-negative bacterial strains (Table 6). MLE was potent as an extract by inhibiting bacterial growth of all tested strains (Gram-positive and Gram-negative) at a concentration of 0.6% (Table 6).

The results of antibacterial activity of different extracts suggested that the antibacterial activity of OLE against Gram-positive bacteria is essentially due to the phenolic compounds as ligestroiside, oleuropein, tyrosol, hydroxytyrosol, etc. [18,69]. These observations are consistent with those reported by Alberto et al. [20]. These authors found that OLE has no antibacterial effect toward *E. coli* and *S. enterica*. In contrast, these results were not consistent with those reported by Liu et al. [55], who found that olive leaf extract (62.5 mg/mL) almost completely prevented the growth of *Listeria monocytogenes, Escherichia coli* O157:H7, and *Salmonella* Enteritidis. However, the antimicrobial activity of FLE against Gram-negative bacteria is partly due to the presence of phenolic compounds, such as caftaric acid, quercitin, *p*.hdroxybenzoic acid, caffeic acid, gallic acid, etc. [70,71]. The results revealed that the FLE was less potent than the OLE against *Staphylococcus aureus* [72]. The results from this study are in agreement with the recent findings reported by Mahmoudi et al. [73], who found that FLE has antimicrobial activity against Gram-negative and Gram-positive bacteria.

The MLE presented a synergistic effect between FLE and OLE (1:1). The antibacterial activity of MLE against both Gram-negative and Gram-positive bacteria is likely due to the combination of phenolic compounds in FLE (caftaric acid, quercitin, *p*. hdroxybenzoic acid, caffeic acid, gallic acid, etc.) and phenolic compounds in OLE (ligestroiside, oleuropein, tyrosol, hydroxytyrosol, etc.). The present study showed that the synergistic effect highly increased antibacterial properties against foodborne pathogens and thermoduric bacteria. In agreement with the present study, Abeed et al. [72] reported the antibacterial activity of FLE and OLE, both separately and synergistically, toward MRSA. The results indicated a slightly synergistic impact against Methicillin-resistant *Staphylococcus aureus* (MRSA).

### 3.7. Antibacterial Effect of FLE, OLE, and MLE on Pasteurized Buffalo Milk Samples

**Figure 2:**
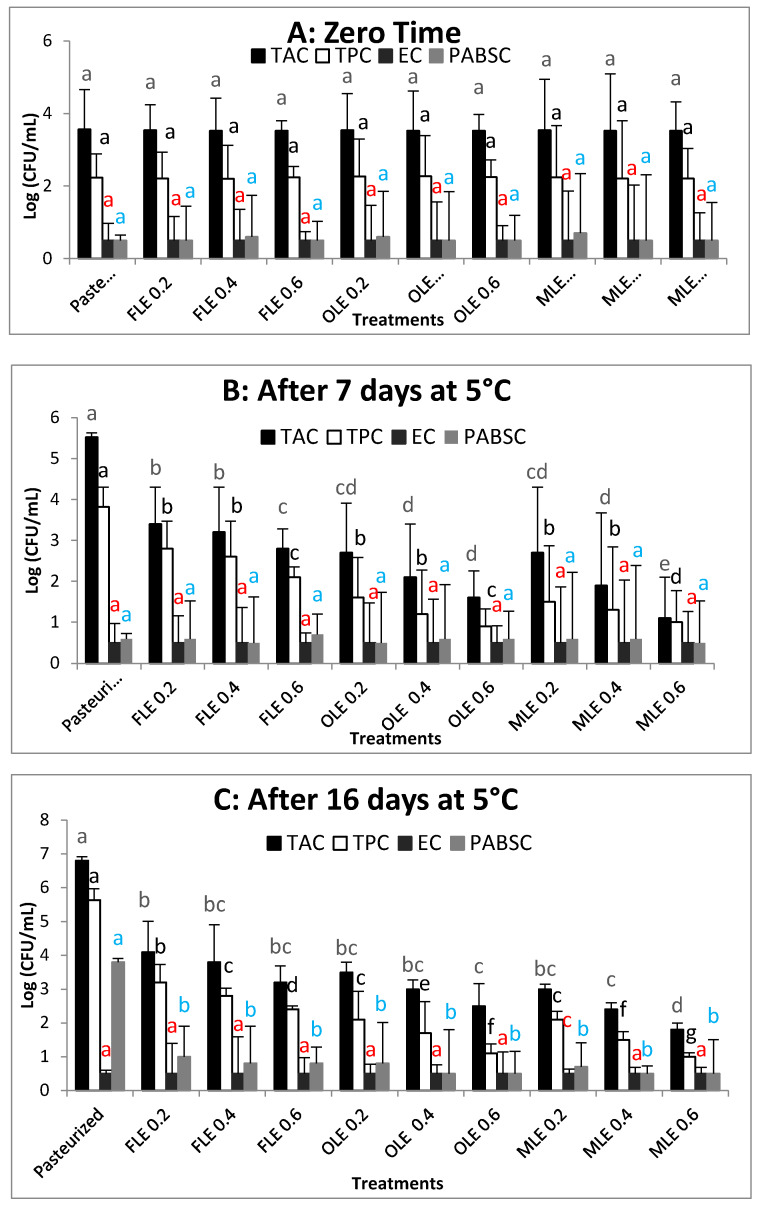
Microbial profile analysis (TAC, TPC, EC, and PABSC) of pasteurized buffalo milk samples enriched with different concentrations of FLE, OLE, and MLE during 16 days of storage at 5 °C. (**A**) zero time, (**B**) after 7 days at 5 °C and (**C**) after 16 days at 5 °C. Columns at the same microbiological parameter (TAC, TPC, EC, and PABSC) are labeled to show significant differences between different treatments by using different letters. Vertical bars show the standard error of the samples mean.

Total coliform count in all treatments was below the limit of detection (LOD) of the plate count procedure over the storage time (16 days). Figure 2 shows the TAC, TPC, and PABSC counts of pasteurized milk samples treated with different concentrations (0.2%, 0.4%, and 0.6%) of OLE, FLE, and MLE during 16 days at 5 °C. The mean value of TCA in pasteurized milk (control) was 3.65 log CFU/mL, with no significant (*p* = 0.05) differences compared with other treatments at zero time (Figure 2A). When the pasteurized milk samples were kept at 5 °C for 16 days, obvious increases were observed in total viable count, particularly when pasteurized milk samples were stored for 16 days (Figure 2A–C). The rate of increase was approximately 2 log CFU/mL after 7 days of storage, whereas the rate of increases was approximately 3.3 log CFU/mL after 16 days of storage (Figure 2A–C). The antibacterial activity of FLE, OLE, and MLE was more efficient with elevated concentrations (Figure 2). In particular, the starting TAC, TPC, and PABSC counts (zero time) were further observed to meet the recommended standards of the European Union [74] and Egyptian standard [75]. After 6 days of storage period (the expiration date of pasteurized milk), the total aerobic count in pasteurized milk without the addition of extracts exceeded the permitted standard of European Union and Egyptian communities [74]. The total aerobic count was 3.4 ± 0.90, 3.2 ± 0.10, and 2.8 ± 0.48 log CFU/mL for pasteurized milk enriched with FLE at 0.2%, 0.4%, and 0.6%, respectively, while the TAC was 2.7 ± 1.2, 2.1 ± 1.3, and 1.6 ± 0.65 log CFU/mL for pasteurized milk containing OLE at 0.2%, 0.4%, and 0.6%, respectively. In addition to 2.7 ± 1.6, 2.4 ± 1.77, and 1.5 ± 1.0 log CFU/mL for the pasteurized milk samples containing MLE at 0.2%, 0.4%, and 0.6%, respectively. These counts steadily increased over storage, reaching 4.1 ± 0.91, 3.8 ± 1.1, and 3.2 ± 0.49 log CFU/mL after 16 days of storage in pasteurized milk samples enriched with 0.2%, 0.4%, and 0.6% of FLE, respectively (Figure 2C). Moreover, the values of TAC in samples containing 0.2%, 0.4%, and 0.6% of OLE steadily increased over storage, reaching 3.5 ± 0.3, 3 ± 0.28, and 2.5 ± 0.66 log CFU/mL, respectively, after 16 days of storage (Figure 2C). However, the total aerobic counts in samples containing 0.2%, 0.4%, and 0.6% of MLE steadily increased over storage, reaching 3 ± 0.15, 2.4 ± 0.2, and 1.8 ± 0.2 log CFU/mL, respectively, after 16 days of storage (Figure 2C). Similar results were observed in the total pyschrotrophs. Particularly, TPC in pasteurized milk samples at zero-time (Figure 2A) presented a mean value of 2.23 log CFU/mL for TPC, with no significant (*p* = 0.05) difference compared with other treatments. Clear increases were observed in TPC, particularly when pasteurized milk samples were kept at 5 °C for 16 days (approximately 3.4 log CFU/mL). Adding the highest concentration (0.6%) of MLE showed a significant (*p ≤* 0.05) difference on TAC and TPC in pasteurized milk at 5 °C after 16 days of storage, compared with other treatments (Figure 2A–C). All concentrations of FLE, OLE, and MLE decreased TAC. The results ranged from 1.8–4.1 log CFU/mL after 16 days, and they fully comply with the mentioned standards European limit (4.7log CFU/mL) [74].

PABSC scarcely existed at detectable limits (Figure 2A–C). This may be attributed to a shorter period of storage time at 5 °C. The mean values of PABSC in pasteurized milk samples were kept at 5 °C for zero time and 7 days of storage period, ranging from 0.5 to 0.6 log CFU/mL, respectively. There was no significant (*p* < 0.05) differences compared with other treatments. Clear increases were observed in PABSC when a pasteurized milk sample (control) was stored for 16 days (approximately 3.2 log CFU/mL). The high concentration (0.6%) of FLE, OLE, and MLE prevented the outgrowth of PABSC after 16 days of storage at 5 °C (Figure 2C). Previous studies have presented that the adverse impacts of PABSC (*Paenibacillus* spp. or *Bacillus* spp.) on quality of pasteurized milk are clearly and consistently elucidated after a long cold period (more than 15 days) and associated with a high reduction of Gram-negative psychrotrophic bacteria that causes post-pasteurization contamination [76]. The current results are consistent with Palmeri et al. [34], who reported that the milk enrichment with olive leaf extract (at 3.6 mg of oleuropein/mL) of pasteurized milk decreased TAC at under detection limit after expiry date (6 days) and by 1 Log CFU/mL after 10 days. Debib, Tir-Touil, Meddah, Hamaidi-Chergui, Menadi, and Alsayadi [27] also reported that the dried figs macerates extract and olive oil extract presented wide spectrum activity against both Gram-negative and Gram-positive bacteria. Several previous studies reported the use of natural preservatives (essential oils, nisin, and their combination) for extending the shelf life of pasteurized milk from 5–10 days to 20–30 days [77].

It is clear from the results in Figure 2A–C that the antimicrobial effect of OLE was more potent than FLE. The mixture of FLE and OLE (1:1) exhibited more synergistic effect at the highest concentration (0.6%) of MLE. These results are in agreement with the data reported by Abeed et al. [72]. These authors found that the combination with ethanol extract of fig and olive leaves with different ratios presented a slightly synergistic antimicrobial effect against Methicillin-resistant *Staphylococcus aureus* (MRSA) [72].

### 3.8. Determination of the pH and the Acidity Level in the Pasteurized Milk over the Storage Period at 5 °C

**Figure 3:**
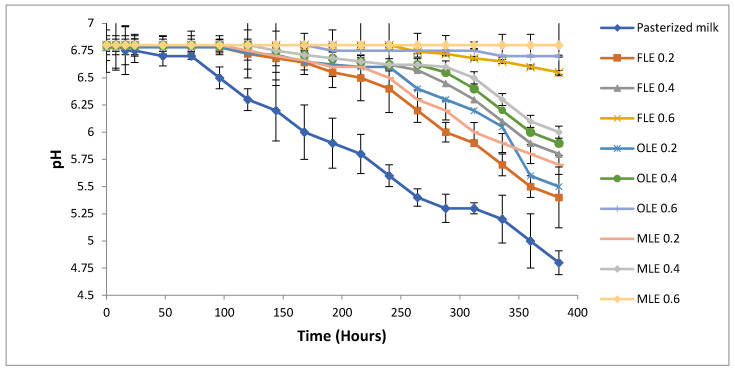
Observed average shelf stability of pasteurized buffalo milk enriched with FLE, OLE, and MLE based upon pH at 5 °C for 16 days. Vertical bars show the standard error of the samples’ mean.

**Figure 4:**
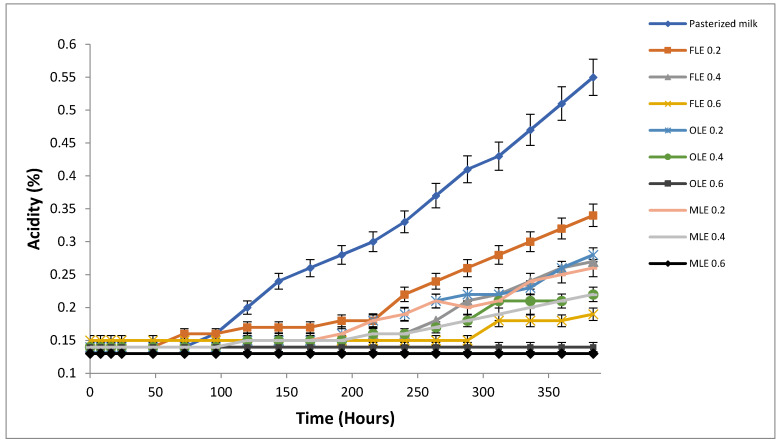
Observed average shelf stability of pasteurized buffalo milk enriched with FLE, OLE, and MLE, based upon titratable acidity at 5 °C for 16 days. Vertical bars show the standard error of the samples’ mean.

The rates of change in the pH and the acidity values of all treatments over the storage period (16 days) at 5 °C were determined (Figure 3 and Figure 4), respectively. The decline of the pH value is related to lactic acid production over storage, which will decrease the shelf life of pasteurized milk. The pH value alone is not considered a credible shelf-life indicator for pasteurized milk, but it can be improved by combining pH and aerobic plate counts [47]. The normal pH value of fresh milk ranged from 6.6 to 6.8 [78] (Figure 3). Some researchers have reported that the pH value is not preferably used to detect shelf life of pasteurized milk [79,80]. However, other scientists employed change in pH to detect validation of pasteurized milk over storage [76,81]. The pasteurized milk (control) sample exceeded the critical pH after 5 days during storage at 5 °C (Figure 3). The shelf life of pasteurized milk samples treated with different concentrations of extracts was extended from 100% to 220%, according to types and concentrations of extracts (Figure 3). All pasteurized milk samples treated with 0.6% of FLE, OLE, and MLE had a shelf life extended until 16 days, while adding 0.4% of extracts extended shelf life to 12–14 days, according to types of extract (Figure 3). The shelf life of pasteurized milk samples treated with 0.2% of extracts reached 10–11 days (Figure 3). The extended shelf life of pasteurized milk was thus directly proportional with the concentrations of the extracts (Figure 3).

The normal acidity in pasteurized milk during shelf life ranged from 0.13% to 0.19% [82]. The percent change in acidity is more highly associated with the degree of fermentation of lactose by the microbial content of pasteurized milk than pH. The increase in titratable acidity in pasteurized milk was found to be directly proportional to the decrease in shelf life of pasteurized milk. Using titratable acidity as an indicator for the pasteurized milk shelf life is better than using pH, because pasteurized milk constituents have a high buffering capacity [47]. The addition of FLE, OLE, and MLE with different concentrations to pasteurized milk has a significant effect on the acidity and subsequently the shelf life of pasteurized milk, (Figure 4). The acidity values for pasteurized samples treated with 0.6% of FLE, OLE, and MLE were low (<0.20%), around that of fresh pasteurized milk, until 16 days of storage at 5 °C. The acidity of pasteurized milk samples treated with 0.4% of FLE, OLE, and MLE at 5 °C exceeded 0.20% value after 12, 13, and 14 days, respectively. However, the rate of increase in the acidity percentage of pasteurized milk samples enriched with 0.2% of FLE, OLE, and MLE exceeded 0.20% after 10, 11, and 12 days, respectively. A sharp increase in acidity of pasteurized milk without the addition of aqueous extracts (control) resulted in an acidity exceeding 0.20% after 5 days. Although TAC did not exceed 5.0 log CFU/mL in almost all samples until 16 days of storage, a gradual increase in acidity was observed in these samples. This might be attributed to the capacity of thermotolerant lactic acid bacteria that are the main factor responsible for lactic acid production in pasteurized milk [83]. Lactic acid bacteria are fastidious and require complex growth factors [84]. Therefore, the TAC does not include lactic acid bacteria. The stability of the acidity of the milk containing 0.6% of different extracts is probably related to the antimicrobial activity of these extracts [85].

### 3.9. Determination of Protease Activity in Pasteurized Buffalo Milk 

**Figure 5:**
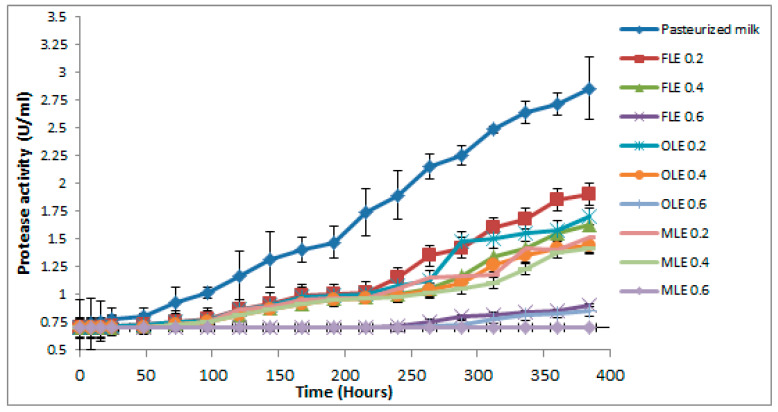
Observed average shelf stability of pasteurized buffalo milk enriched with FLE, OLE, and MLE, using activity of protease at 5 °C for 16 days. Vertical bars show the standard error of the samples mean.

Proteolysis is produced by psychrotrophic microorganisms that grow at low temperature (≤7 °C) [86]. During the storage of pasteurized milk at 5 °C, these microorganisms play a critical role in the microflora formation and are responsible for several problems in pasteurized milk [86]. In good hygiene practices, the percentage of psychrotrophic microbial is lower than 10% of the total microbial flora in pasteurized milk. In comparison, the percentage would increase to up to 75% in poor hygiene practices [87]. The extracellular proteases are highly heat stable and active over a broad range of pH and temperature [88]. The results in Figure 5 revealed that pasteurized milk treated with high concentrations (0.6%) of FLE, OLE, and MLE significantly decreased (*p < 0.05*) growth of TPC and lowered protease activity (Figure 5).

The pasteurized milk (control) sample exceeded the critical protease activity level after 5 days storage at 5 °C (Figure 5). All pasteurized buffalo milk samples treated with 0.6% of FLE, OLE, and MLE did not exceed the critical protease activity level until 16 days, while 0.4% of extracts exceeded the critical point of protease activity after 12–14 days, according to types of extract (Figure 5). However, the critical level of protease activity in pasteurized milk samples treated with 0.2% of extracts was reached after 10–11 days (Figure 5).

### 3.10. Determination of Lipolysis Activity in Pasteurized Milk

**Figure 6:**
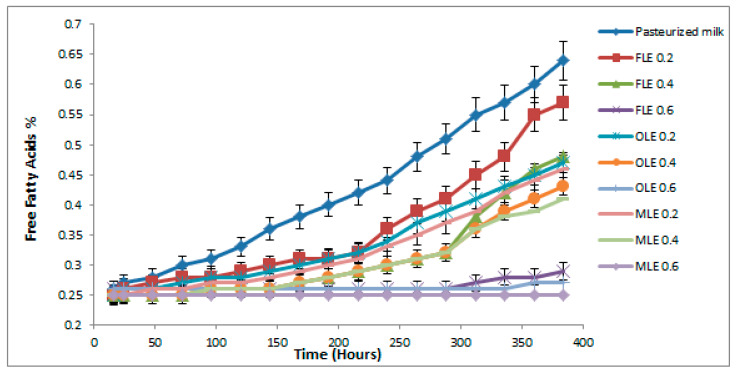
Observed average shelf stability of pasteurized buffalo milk enriched with FLE, OLE, and MLE, using free fatty acids at 5 °C for 16 days. Vertical bars show the standard error of the samples mean.

Free fatty acids (FFA) were utilized as a lipolysis indicator. In this study, free fatty acids percentage was used as a significant indicator for pasteurized milk spoilage (Figure 6). Some Gram-negative bacteria strains that tolerate pasteurization can secrete extracellular enzymes that are effective at low temperatures. Although these microorganisms may be destroyed, the residual thermal stability enzymes may hold up to 74% of their initial effective after heat stress [89,90].

The pasteurized milk (control) sample exceeded the critical FFA level after 5 days during storage at 5 °C (Figure 6). All pasteurized milk samples treated with 0.6% of FLE, OLE, and MLE did not exceed the critical FFA level until 16 days. Pasteurized milk treated with 0.4% of extracts exceeded the critical point of FFA after 12 days (Figure 6). However, the critical level of FFA in pasteurized milk samples treated with 0.2% of extracts reached the critical FFA percent after 10 days (Figure 6).

### 3.11. Sensory Analysis of Pasteurized Buffalo Milk Enriched with FLE, OLE, and MLE

**Figure 7:**
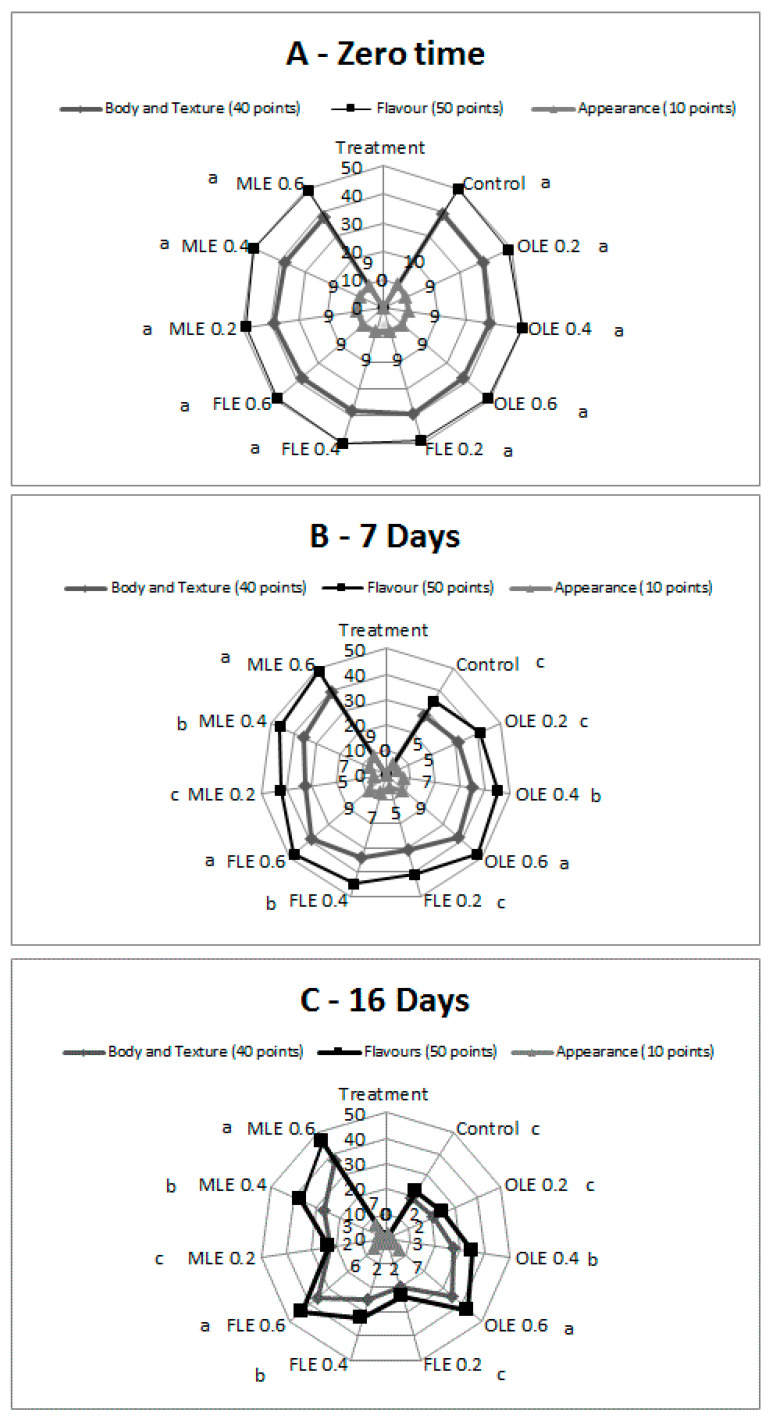
Sensory profile analysis of pasteurized buffalo milk samples enriched with different concentrations of FLE, OLE, and MLE, during 16 days of storage, at 5 °C. (**A**) zero time, (**B**) after 7 days at 5 °C and (**C**) after 16 days at 5 °C Significant differences between different treatments are labeled significant differences between different treatments by using different letters.

The sensory properties of ten milk samples were evaluated to determine the influence of FLE, OLE, and MLE on sensory characteristics (Figure 7). The results of the sensory evaluation indicate that the total score of sensory properties for all pasteurized milk samples was similar, ranging from 98% to 99% at zero time. There were not significant differences (*p* < 0.05) in sensory scores between control pasteurized milk and the other samples containing different concentrations of FLE, OLE, and MLE, suggesting that the addition of FLE, OLE, and MLE with the highest concentration did not alter the sensory score at zero time (Figure 7A), while the sensory analysis after 7 days of storage at 5 °C indicates that the total score was ranging from 68% to 97% for pasteurized milk and pasteurized milk enriched with 0.6% of MLE, respectively (Figure 7B). Using the highest concentration of FLE, OLE, and MLE significantly increased the sensory score at 7 days of storage at 5 °C. However, the sensory characteristics after 16 days of storage at 5 °C showed that the total score was ranging from 43% to 90% for pasteurized milk and pasteurized milk enriched with 0.6% of MLE, respectively (Figure 7C). The addition of the highest concentration (0.6%) of FLE, OLE, and MLE significantly increased the total score at 16 days of storage at 5 °C. These results are consistent with Palmeri et al. [34], who found that the addition of OLE (5%) did not affect the sensory properties of pasteurized buffalo milk.

## 4. Conclusions

The purpose of this study was to test the possibility of extending the shelf life of pasteurized milk from 5 to 16 days, using different concentrations (0.2%, 0.4%, and 0.6%) of OLE, FLE, and MLE. The results of this study proved that using the high concentration (0.6%) of MLE was more effective than using either OLE or FLE with the same concentration. This suggests a synergistic effect of FLE and OLE (MLE) that leads to an increase in the antibacterial activity against bacterial food infection (*E. coli*, *S*. Typhi, and *P. aeruginosa*), bacterial food poisoning (*Staph. aureus* and *Bacillus cereus*), and thermoduric bacteria (*En. faecalis)*. The highest polyphenol content of 387 mg of gallic acid/g of extract was found in the olive extract, compared with 224 mg of gallic acid/g of extract in fig extract. It can be concluded that the antioxidant activity was higher in OLE than in FLE. Caftaric acid was characterized as the main phenolic compound in FLE, while oleuropein was identified as the primary phenolic compound in OLE. The highest concentration (0.6%) of MLE presented significant (*p ≤* 0.05) antibacterial activity on TAC, TPC, and PABSC in pasteurized milk after 16 days of storage at 5 °C. All pasteurized milk samples treated with 0.6% of FLE, OLE, and MLE did not exceed the critical protease and lipase activity level, in addition to the stability of acidity, until 16 days. It can be concluded that extending the shelf life of pasteurized buffalo milk from 5 to 16 days was successfully achieved by using 0.6% of FLE, OLE, and MLE. The combination of the two extracts (MLE) provides a useful and safe method for prolonging the shelf life of pasteurized milk, without altering the properties of pasteurized buffalo milk.

## Figures and Tables

**Table 1:** Chemical composition of fig and olive leaves on dry-weight basis.

Leaves	Moisture %	Ash %	Crude Protein %	Crude Lipid %	Crude Fiber %	Total Carbohydrates %	Soluble Carbohydrates %
**Olive**	8.12 ± 0.15	2.87 ± 0.22	12.38 ± 0.2	3.12 ± 0.34	27.73 ± 0.36	53.90 ± 0.24	26.17 ± 0.36
**Fig**	9.57 ± 0.11	1.66 ± 0.19	7.22 ± 0.18	2.08 ± 0.31	30.81 ± 0.26	58.23 ± 0.76	27.42 ± 0.11

The data presented are the mean of three replicates ± standard error.

**Table 2:** Minerals composition of fig and olive leaves (mg/100 g) on dry weight (DW) basis.

Leaves	Minerals Content (mg/100 g DW)					
	Calcium	Phosphorus	Iron	Potassium	Magnesium	Manganese
**Olive**	1570 ± 0.14	120 ± 0.18	19.1 ± 0.21	660 ± 0.11	200 ± 0.20	4.3 ± 0.26
**Fig**	1400 ± 0.36	365.97 ± 0.13	115.3 ± 0.41	118.47 ± 0.27	400 ± 0.13	22.6 ± 0.36

The data presented are the mean of three replicates ± standard error.

**Table 3:** Preliminary phytochemical screening of fig and olive leaf aqueous extract.

Aqueous Leaves Extract	Flavonoids	Steroids	Tannins	Saponins	Alkaloids	Glycosides
**Olive**	+ *	+	+	−	+	+
**Fig**	+	+	+	+	+	+

* The data are recorded as visual observation, where “+” means that the presence of the component is detected, and “−” means that the component is not detected.

**Table 4:** Total phenolic compound contents (mg/g) and antioxidant activity (IC50) of fig and olive leaves’ extract.

Leaves	Total Phenolic (mg of Gallic Acid Equivalent/g Extract)	Antioxidant Activity (IC_50_) µg/mL
**Fig**	224.33 ± 0.55	30.21 ± 0.54
**Olive**	387.00 ± 0.55	22.43 ± 0.54

The data presented are the mean of three replicates ± standard deviation.

**Table 5:** HPLC analysis of extracts of the phenolic compounds of FLE and OLE.

Entry	Compound	Fig Leaves Extract (mg/g Dried Extract)	Olive Leaves Extract (mg/g Dried Extract)
**1**	Pyrogallol	0.006	0.005
**2**	Quinol	0.011	-
**3**	Gallic acid	1.5	0.029
**4**	*p*-Hydroxy benzoic acid	3.5	-
**5**	Chlorogenic acid	0.002	0.03
**6**	Vanillic acid	0.079	-
**7**	Caffeic acid	2.48	0.032
**8**	Syringic acid	0.097	0.005
**9**	*p*-Coumaric acid	0.013	0.018
**10**	Ferulic acid	0.032	0.015
**11**	Benzoic acid	0.32	0.128
**12**	Caftaric acid	40.2	-
**13**	Ellagic acid	0.524	0.269
**14**	*o*-Coumaric acid	0.011	-
**15**	Salicylic acid	0.045	0.040
**16**	Myricetin	0.414	0.131
**17**	Oleuropein	-	32.2
**18**	Quercitin	13.4	0.218
**19**	Rosmarinic acid	0.270	-
**20**	Ligstroside	0.188	4.2
**21**	Kampherol	0.88	0.32

**Table 6:** The antimicrobial activity of OLE, FLE, and their mixture against *P. aeruginosa*, *S*. Typhi, *Staph. aureus, E. coli, E. faecalis,* and *B. cereus*.

Treatments	Concentration (%)		Inhibition Zone (mm)				
		*P. aeruginosa*	*Salmonella* Typhi	*Staphylococcus aureus*	*Escherichia coli*	*Enterococcus facials*	*Bacillus cereus*
FLE	0.2	16 ± 0.11 ^d^	18 ± 0.54 ^c^	14 ± 0.41 ^e^	13 ± 0.13 ^c^	5 ± 0.22 ^d^	5 ± 0.11 ^d^
	0.4	21 ± 0.07 ^cd^	20 ± 0.23 ^c^	15 ± 0.15 ^e^	14 ± 0.09 ^c^	7 ± 0.13 ^d^	5 ± 0.16 ^d^
	0.6	24 ± 0.17 ^c^	22 ± 0.27 ^bc^	17 ± 0.13 ^de^	18 ± 0.16 ^b^	7 ± 0.19 ^d^	6 ± 0.21 ^d^
OLE	0.2	5 ± 0.31 ^e^	4 ± 0.29 ^e^	19 ± 0.35 ^d^	5 ± 0.21 ^d^	19 ± 0.32 ^c^	21 ± 0.31 ^c^
	0.4	6 ± 0.22 ^e^	6 ± 0.19 ^de^	24 ± 0.12 ^c^	7 ± 0.24 ^d^	23 ± 0.41 ^bc^	27 ± 0.45 ^b^
	0.6	6 ± 0.41 ^e^	8 ± 0.27 ^d^	28 ± 0.38 ^b^	8 ± 0.51 ^d^	26 ± 0.25 ^b^	29 ± 0.13 ^b^
MLE	0.2	27 ± 0.45 ^b^	24 ± 0.43 ^b^	27 ± 0.46 ^b^	20 ± 0.19 ^b^	27 ± 0.11 ^ab^	29 ± 0.22 ^b^
	0.4	29 ± 0.42 ^b^	25 ± 0.14 ^b^	30 ± 0.64 ^a^	22 ± 0.26 ^b^	29 ± 0.36 ^a^	32 ± 0.18 ^ab^
	0.6	33 ± 0.15 ^a^	30 ± 0.61 ^a^	32 ± 0.71 ^a^	26 ± 0.23 ^a^	30 ± 0.31 ^a^	34 ± 0.21 ^a^

* a–e: statistical analysis as the mean comparison; Means with the same letter in a column are not significantly different at p > 0.05.

## Supplementary Materials

The following are available online at https://www.mdpi.com/2304-8158/9/5/615/s1. Table S1: The characteristics and performances of the calibration curves of the individual reference standards.
