# Supplementary material for: Potential Activity of Aqueous Fig Leaves Extract, Olive Leaves Extract and Their Mixture as Natural Preservatives to Extend the Shelf Life of Pasteurized Buffalo Milk
Source: Foods. 2020 May 11;9(5):615. doi: 10.3390/foods9050615 (PMC7278658; doi:10.3390/foods9050615)
Supplement: Supplementary file 1 [file foods-09-00615-s001.pdf]

Table S1. The characteristics and performances of the calibration curves of the individual reference standards.

	**Reference standard**	**Regression equation**	**Correlation coefficient**	**LOD/μg mL^{-1}**	**LOQ/μg mL^{-1}**
1	Pyrogallol	y=3.894x–3.485	0.9905	0.0028	0.0034
2	Quinol	y = 12.833x – 4.0785	0.9941	0.005	0.009
3	Gallic acid	y = 25.884x – 95.518	1.000	0.012	0.018
4	*p*-Hydroxy benzoic acid	y=6.241x–10.241	0.9982	1.421	1.834
5	Chlorogenic acid	y=4.531x–8.725	0.9902	0.0012	0.0015
6	Vanillic acid	y=35.217x–41.481	1.000	0.032	0.058
7	Caffeic acid	y=5.391x–12.834	1.000	0.014	0.022
8	Syringic acid	y=41.857x–20.321	0.995	0.0019	0.0024
9	*p*-Coumaric acid	y = 26.432x – 18.28	0.9923	0.006	0.009
10	Ferulic acid	y = 29.836x – 22.876	0.9961	0.009	0.012
11	Benzoic acid	y = 6.246x – 0.763	0.9903	0.118	0.122
12	Caftaric acid	y=42.168x–17.416	0.9914	2.632	2.853
13	Ellagic acid	y = 31.472x – 5.835	1.000	0.175	0.192
14	*o*-Coumaric acid	y = 24.423x – 8.172	0.9908	0.003	0.005
15	Salicylic acid	y = 35.451x – 23.451	1.000	0.024	0.032
16	Myricetin	y=11.485x–6.184	0.9972	0.042	0.096
17	Oleuropein	y=2.579x–1.249	1.000	3.172	3.451
18	Quercitin	y = 1.5823x – 19.450	1.000	0.126	0.149
19	Rosmarinic acid	y = 9.814x – 3.0758	0.9943	0.178	0.216
20	Ligstroside	y=1.324x-4.124	1.000	0.112	0.118
21	Kampherol	Y=19.346x-6.568	1.000	0.15	0.24
